# The characteristics of circular disposable devices and *in situ* devices for optimizing male circumcision: a network meta-analysis

**DOI:** 10.1038/srep25514

**Published:** 2016-05-09

**Authors:** Yu Fan, Dehong Cao, Qiang Wei, Zhuang Tang, Ping Tan, Lu Yang, Liangren Liu, Zhenhua Liu, Xiang Li, Wenbin Xue

**Affiliations:** 1 Department of Urology, Institute of Urology, West China Hospital of Sichuan University, Chengdu, China; 2 Department of Urology, Mianyang central hospital, Mianyang, China

## Abstract

*In situ* device (ISD) and circular disposable device (CDD) are used for optimizing male circumcision (MC), but evidence to explore the characteristics of these two devices is insufficient. In order to explore this issue systematically and provide reliable evidence, ten published randomized controlled trials (RCTs) exploring the safety and efficacy of ISDs and CDDs were included (involving 4649 men). Moderate quality of the RCTs included was found after assessment. Pairwise meta-analyses and network meta-analyses were processed in stata 13.0 and AIDDS v1.16.6 respectively. According to the outcomes that were statistically significant in both pairwise and network meta-analyses, ISD was found to have less intraoperative blood loss (IB), less operative time (OT) and less incidence of wound bleeding (WB) than conventional circumcision (CC); ISD was found to have less WB but more wound healing time (WHT) than CDD; CDD was found to have less IB and less OT than CC. CDD tended to have the best wound healing condition and least pain experience; ISD tended to have the least IB, least OT, least WB, and highest satisfaction rate. With their own superiorities in many aspects, CDD and ISD are both safe and effective devices for optimizing MC.

Male circumcision (MC) is the most commonly performed surgical procedure in the world^1^ and has demonstrated its effectiveness on reducing various sexually transmitted infections, such as human immunodeficiency virus, human papillomavirus, and herpes simplex virus 2^2^,^3^,^4^,^5^. Moreover, it is associated with the prevention of urinary tract infections, penile cancer, prostate cancer, and cervical cancer of female partners^6^,^7^,^8^,^9^,^10^.

Conventional circumcision (CC) is recommended by World Health Organization (WHO) as the standard procedure to remove the redundant prepuce^11^. However, CC has some limitations: it is time consuming, painful, requires stitches, and its operative incision visibility lacks controllability^12^,^13^. In addition, adequate training (an average of 100 circumcisions) is required for practitioners to perform CC safely and effectively^14^. To facilitate the surgical procedures, promote the recovery process, and satisfy the penile appearance, several special disposable devices with mature surgical methods are being routinely used in many medical centers^11^.

Pediatric and neonatal MCs are often performed using one of the several different clamping or crimping devices, such as Plastibell, Gomco, Mogen, or AccuCirc^15^,^16^,^17^. For adult MC, Shang Ring and PrePex, two *in situ* devices (ISD), have been proven to have better clinical outcomes than CC. They are associated with a shorter operative time (OT), a lower intraoperative pain score, a higher satisfaction with penile appearance, less intraoperative blood loss (IB), a lower rate of adverse event, and a lower incidence of wound bleeding (WB)^13^,^18^,^19^. Circular disposable devices (CDD), such as the Circular Stapler^20^ and Unicirc^12^, are based on a principle different from that of ISD and may be superior to CC^21^. However, credible evidence is insufficient to confirm whether ISD or CDD can optimize MC, and comprehensive comparisons of their advantages and disadvantages are lacking. Therefore, we conducted this systematic review and network meta-analysis to assess the safety and efficacy of ISDs and CDDs. Furthermore, we evaluated the characteristics of these devices for optimizing MC.

## Results

### Selected trials

**Figure 1:**
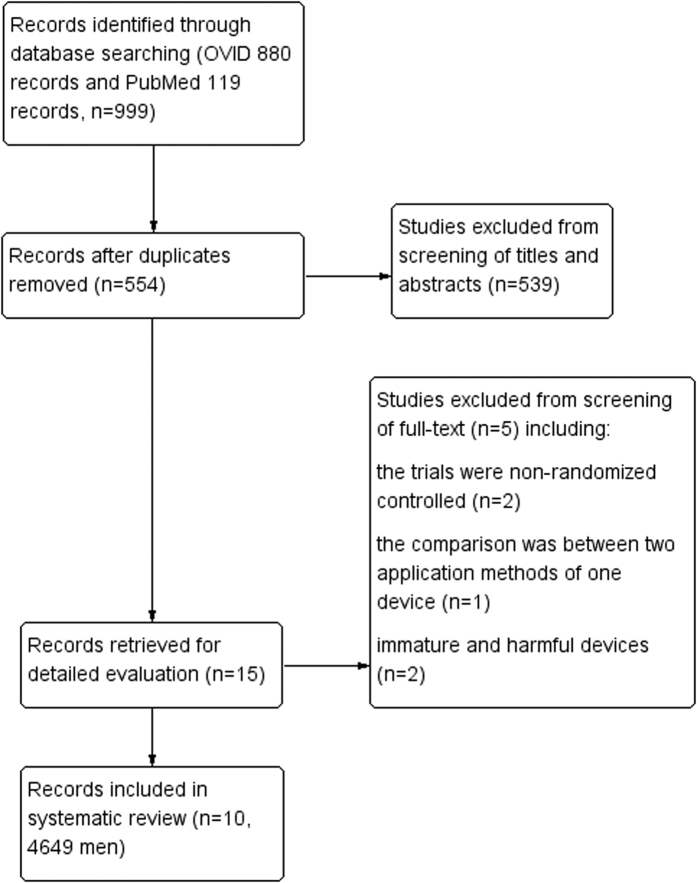
Flow diagram of systematic review.

**Figure 2:**
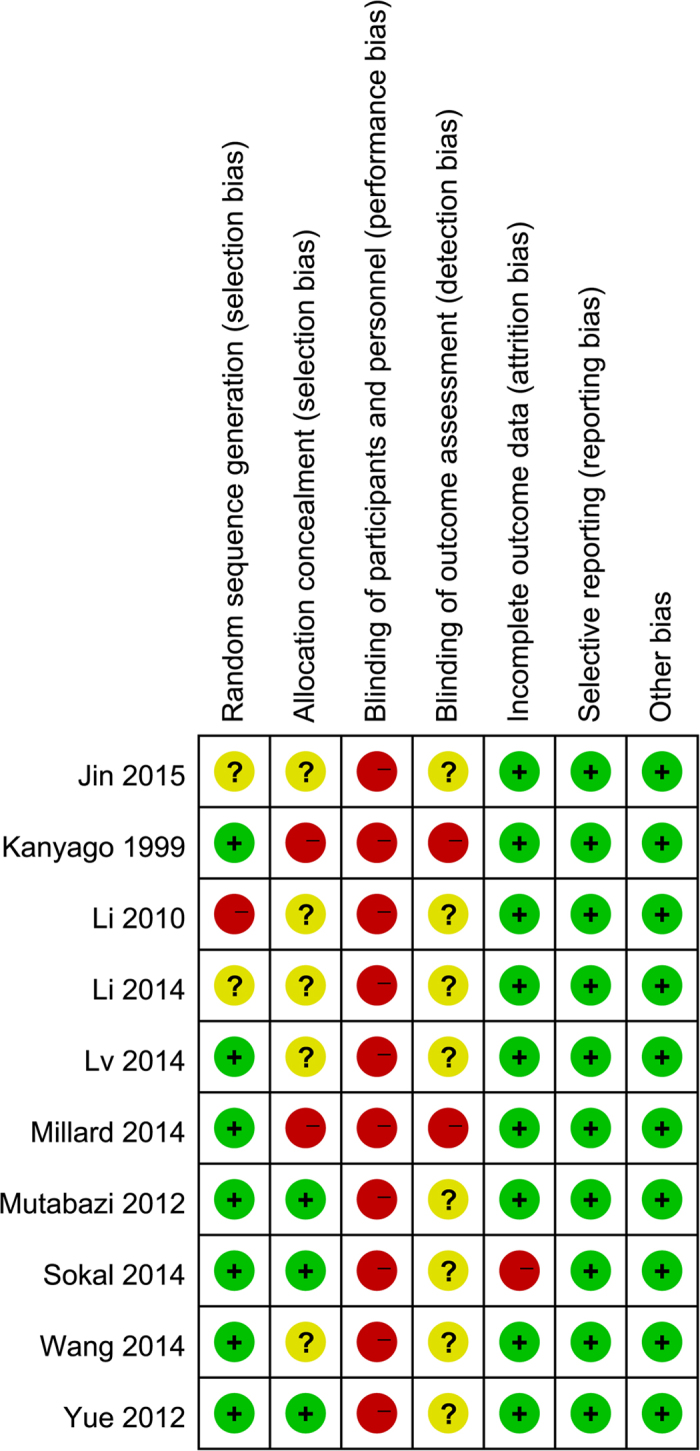
Risk of bias summary of included studies. (+: Low risk of bias; ?: Unclear risk of bias; -: High risk of bias)

Pubmed and Ovid databases were searched and 491 and 838 references were evaluated, respectively. After 775 duplicates were removed, the titles and abstracts of 554 records were read, and 539 records that did not meet the inclusion criteria were excluded. Fifteen trials were retrieved for a detailed evaluation and five were excluded: two non-randomized controlled trials, one trial that compared two application methods of one device, and two trials that used immature^15^ and harmful devices^22^. Ten randomized controlled trials (RCTs) were identified (4649 men)^12^,^18^,^20^,^21^,^23^,^24^,^25^,^26^,^27^,^28^ and included in our meta-analysis (Fig. 1); moderate methodological quality is shown in Fig. 2. Seven trials^12^,^18^,^21^,^23^,^26^,^27^,^28^ had a low risk of bias of a random sequence generation; only three trials^18^,^26^,^28^ had a low risk of allocation concealment; and none of the trials had a low risk of performance bias or detection bias. However, there was a low risk of attrition bias, reporting bias, and other biases in all trials except one^26^ that had an incomplete outcome data.

**Figure 3:**
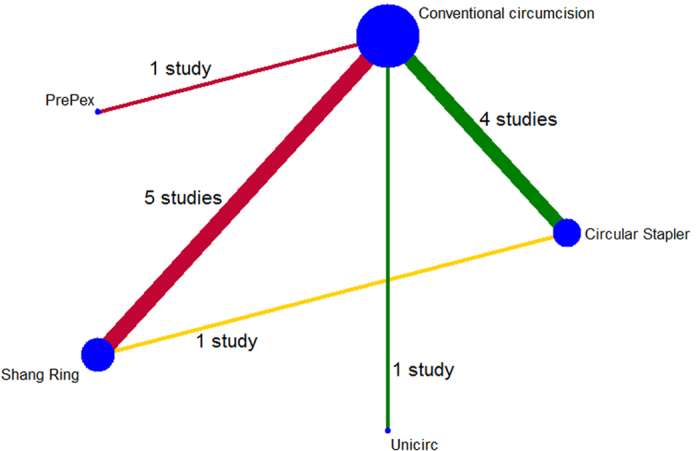
Network plot of the circumcision. Both nodes and edges are weighted according to the number of studies involved in each direct comparison. Green line: CDD vs CC; Red line: ISD vs CC; Yellow line: CDD vs ISD. (CDD: circular disposable devices, ISD: *in situ* devices, CC: conventional circumcision)

Of all the RCTs, six were conducted in China^20^,^21^,^24^,^25^,^27^,^28^, and the remaining four were conducted in South Africa^12^, Uganda^23^, Rwanda^18^, and Kenya and Zambia^26^. Nine RCTs were two-arm trials: circular stapler versus CC (three studies), Shang Ring versus CC (four studies), Unicirc versus CC (one study), and PrePex versus CC (one study). One RCT was a three-arm trial: circular stapler versus Shang Ring versus CC. More RCT characteristics are shown in Table 1. The general network situation of eligible comparisons in this meta-analysis is shown in Fig. 3. Five studies compared CDD versus CC, six compared ISD versus CC, and one compared CDD versus ISD directly. However, the numbers of comparisons were variable and less than ten in each of the analyzed outcomes (Tables 2 and 3). Thus, we considered the publication bias in each comparison.

### Credibility of network meta-analysis

We had adequate iterations in each model to make sure every Markov chain was similar and that all potential scale reduction factors (PSRF) were close to 1 (the details are not shown). This ensured that every model in this network meta-analysis converged. Consistency and inconsistency analyses were applied in each outcome and no great difference between random effects standard deviation (RESD) and inconsistency standard deviation (ICSD) was found (Table 4). As comparison involved both direct and indirect evidence of CDD versus ISD, node-splitting models were estimated on the outcomes of this comparison (Table 4). We found that most of evidence was exchangeable, except the WB.

### Comparisons between CDD and CC

Five studies involving 2026 men were included in pairwise meta-analyses (Table 2). The statistically significant outcomes were IB, OT, mean pain score on the operation day (PO), mean pain score on postoperation days (PP), and wound healing time (WHT). CDD showed less IB [standard mean difference (SMD): −3.12 (−4.32, −1.92)], less OT [SMD: −4.33 (−6.43, −2.23)], less WHT [SMD: −0.88 (−1.18, −0.58)], less PO [SMD: −1.51 (−2.55, −0.46)], and less PP [SMD: −1.38 (−2.28, −0.48)] compared with CC.

In network meta-analyses (Table 3), CDD showed less IB [SMD: −8.24 (−11.6, −4.86)], less OT [SMD: −17.3 (−22.6, −11.4)] but a higher overall expenditure (Cost) [SMD: 245 (84.1, 404)] than CC. The stable outcomes with statistical significance in both pairwise and network meta-analyses were for IB and OT.

### Comparisons between ISD and CC

Five studies involving 2937 men were included in pairwise meta-analyses (Table 2). The statistically significant outcomes were IB, OT, satisfaction rate (SR), WB, and incidence of wound edema (WE). ISD showed less IB [SMD: −3.25 (−3.65, −2.85)], less OT [SMD: −5.72 (−7.11, −4.33)], a higher SR [risk ratios (RR): 1.17 (1.02, 1.35)], less WB [RR: 0.16 (0.03, 0.76)], and WE [RR: 0.69(0.53, 0.88)] compared with CC.

In network meta-analyses (Table 3), ISD showed less IB [SMD: −9.26 (−13.5, −4.93)], less OT [SMD: −18.2 (−23.7, −12.6)], and less WB [odds ratio (OR): 0.07 (0, 0.78)] than CC. The stable outcomes with statistical significance in both pairwise and network meta-analyses were for IB, OT, and WB.

### Comparisons between CDD and ISD

Only one study involving 628 men was included in pairwise meta-analyses (Table 2). The statistically significant outcomes were observed for IB, OT, PO, PP, SR, incidence of wound adverse event (WAE), WB, WE, WHT, and incidence of wound infection (WI). CDD showed more IB [SMD: 0.33 (0.17, 0.48)], more OT [SMD: 0.48 (0.32, 0.63)], less PO [SMD: −2.23 (−2.43, −2.03)], less PP [SMD: −2.39 (−2.59, −2.18)], a higher SR [RR: 1.57 (1.39, 1.78)], less WAE [RR: 0.30 (0.20, 0.45)], more WB [RR: 21.0 (1.24, 357)], less WE [RR: 0.10 (0.05, 0.24)], less WHT [SMD: −0.74 (−0.90, −0.58)], and less WI [RR: 0.04 (0.002, 0.62)] compared with ISD.

In network meta-analyses (Table 3), CDD had a higher Cost [SMD: 235 (4.91, 473)] and more WB [SMD: 28.6 (1.17, 1320)] but less WHT [SMD: −6.58 (−12.6, −0.58)] than ISD. The stable outcomes with statistical significance in both pairwise and network meta-analyses were for WB and WHT.

### The surface under the cumulative ranking curve and treatment ranks (Table 5)

The outcomes of CDD whose surface under the cumulative ranking curve (SUCRA) was ≥ 80% were PO, PP, WE, WHT, and WI and whose SUCRA was ≤ 20% were Cost and WB. CDD had a 72%, 73%, 90%, 91%, and 70% possibility for the least PO, PP, WE, WHT, and WI, respectively; CDD had a 97% and 74% possibility to have the highest Cost and WB, respectively.

The outcomes of ISD whose SUCRA were ≥ 80% were IB, OT, SR, and WB and whose SUCRA were ≤ 20% was WHT. ISD had a 70%, 62%, and 77% possibility to have the least IB, OT, and WB, respectively, and a 97% possibility to have the highest SR; ISD had a 95% possibility to have the longest WHT.

No CC outcome had SUCRA ≥ 80%. The outcomes whose SUCRA were ≤ 20% were IB, OT, PO, PP, WI, and SR. CC had a 100%, 85%, 100%, 79%, and 71% possibility to have the largest IB, OT, PO, PP, and WI and a 73% possibility to have the lowest SR.

Although direct and indirect evidence were unexchangeable when comparing WB of CDD and ISD, a sensitivity analysis was applied to explore the reliability of the ranks’ order, and we found that the conclusion was stable (the details are not shown).

## Discussion

The WHO and Joint United Nations Programme on HIV/AIDS (UNAIDS) recommend a voluntary medical MC to be considered as a part of a comprehensive HIV prevention package in countries with generalized epidemics. MC has been proven to have the greatest public health impact and provide the largest cost efficiency if the services are rapidly scaled up. One efficacious way includes using disposable devices in MC; this may lower the surgical skill required and accelerate the pace of delivery of voluntary medical MC while maintaining the safety of the procedure^11^,^19^.

To our knowledge, this is the first network meta-analysis concerning disposable devices in MC. Although our analysis was based on ten studies, it included 4649 individuals who were randomly assigned to three different kinds of MC methods. These trials were conducted in China and some countries in Africa. The methodological quality of RCTs was moderate due to inadequacies in allocation concealment and blinding because of ethical issues and properties of the surgical studies.

In our meta-analysis, devices were classified into one of two categories: ISD (PrePex and Shang Ring) and CDD (circular stapler and Unicirc), according to their operation principles. An ISD consists of an inner and an outer ring. The inner ring is a frame that the outer ring can lock onto to clamp the foreskin^26^,^29^. Excess foreskin is removed immediately after the rings are locked firmly^23^ or at the time when the rings are removed^29^. Rings are removed according to surgeon’s assessment of whether ischemic foreskin has necrosed and the wound has healed. Without a ring *in situ*, CDD has a circular glans pedestal in which the excess foreskin can be incised smoothly and a fastening part to fix the reserved foreskin to prevent shifting. The wound is stapled simultaneously^21^ when incising the foreskin or glued with some type of biogel^12^. The staples will theoretically fall off when the wound has healed.

Some trials involving ISD in Africa were reviewed by the Technical Advisory Group on Innovations in Male Circumcision of the WHO. The outcomes based on a total of 1983 Shang Ring and 2417 PrePex procedures found that ISD was easier to perform, had higher surgical success rates, lower total procedure times, eliminated the need for suturing, possibly had fewer complications, caused less bleeding, gave better cosmetic results, may potentially reduce the time taken for recovery after surgery, and eliminated the need for routine injectable anesthesia (only PrePex) compared with other methods. However, wound healing took about 1–2 weeks longer on average than CC and the pain varied by the method type^19^. We had previously conducted a meta-analysis of RCTs that compared the safety and efficacy of Shang Ring with CC and drew similar conclusions^13^.

The superiority of disposable devices was also confirmed in our meta-analysis. According to the stable outcomes, ISD was found to have less intraoperative blood loss, a lower operative time, and a lower incidence of wound bleeding than CC. In addition, ISD showed a lower incidence of wound bleeding but needed more wound healing time than CDD. CDD was found to have lesser intraoperative blood loss and a lower operative time than CC. After assessing the results of SUCRA and treatment ranks, we found that CDD tended to be the treatment with the best wound healing condition (least incidence of wound edema/infection and wound healing time) and the least pain experience (lowest pain score). However, it may be the most expensive device with the highest incidence of wound bleeding after surgery; among all the techniques, the ISD circumcision tended to have the lowest operative time and bleeding volume intraoperatively and the lowest incidence of bleeding postoperatively. Furthermore, ISD had the highest satisfaction rate despite requiring the longest wound healing time relative to the other techniques. CC showed no advantages other than a minor trend to be the cheapest MC method (78.5% SUCRA, 57% possibility).

Lv *et al.*^21^ conducted a survey on 508 men who had recently undergone circumcision. They found that safety and pain were their main concerns before MC, while pain and penile appearance were their main postoperative concerns. Pain levels varied based on different levels of individual tolerance, methods of anesthesia and analgesia, wound condition (e.g., infection or edema would result in more pain) and so on. In our analysis, both CDD and ISD demonstrated shorter operative times than CC, which was a result of a fast excision of excess foreskin (or no excision at all) and avoiding suturing. It could also be the reason for their lower intraoperative pain score. CDD was also likely to have a better wound condition and that could be the reason for its lower postoperative pain and fast wound healing. Men who chose ISD circumcision were required to wear the ring for 1–2 weeks longer, and pain during an erection was reported as being somewhat higher than at comparable times following a CC (Shang Ring, in particular). However, ISD had a 97% possibility to be the device that best satisfied the men in our analysis. ISD left a neat circumferential wound with no suture marks six weeks postoperatively^19^. However, staples and stitches are used to anastomose foreskin in circular stapler circumcision and CC, respectively, and the unaesthetic appearance may be derived from the imprints and pinholes.

Without a doubt, the most basic and important requirement of safety of CDD and ISD have been proven in many studies and in this meta-analysis. Nevertheless, devices still have their own specific adverse events, such as a dislocation of the frenulum and wound disruption associated with all devices, inconvenience associated with ISD, unpleasant odor associated with PrePex, and potential intraoperative suturing requirement associated with CDD because of bleeding or incomplete wound closure^11^,^12^,^19^,^25^,^29^. We could not explore these issues in our analysis due to lack of adequate numbers of included RCTs.

There are some limitations in our study: We classified devices into two categories but differences still existed within the same category. For example, Shang Ring requires a sterile field and injection of local anesthetic at placement, whereas PrePex requires neither a sterile field nor an injection of local anesthetic at placement. We failed to contact missing data and had a limited number of studies (ten RCTs), and only one comparison between CDD and ISD implies the possibility of a publication bias. Some of the outcomes measured in this meta-analysis were only based on two studies. Studies often report their outcomes in different ways, such as follow-up periods, pain score, and definition of complications. Therefore, all of the results from our meta-analysis should be considered with caution.

In conclusion, the clinical performance of disposable devices used in adult MC exceeded that of CC. CDD circumcision tends to have the best wound healing condition and the least pain experience. ISD circumcision tends to have the lowest operative time, least intraoperative blood loss, least incidence of wound bleeding, and highest satisfaction rate. Each device has its own advantages and these should be discussed with men prior to their circumcision.

## Material and Methods

### Search strategy

A systematic bibliographic search of PubMed, Embase, and the Cochrane Library databases (the Cochrane Central Register of Controlled Trials and the Cochrane database of Systematic Reviews) of Ovid was done from inception to 12 January 2016 for RCTs that reported using disposable devices to complete adult MCs. The following were the keywords used for searching: “circumcision,” “randomized controlled trial” and their other form of expressions. Original papers were scanned in the reference section to look for missing trials.

### Study eligibility criteria

There was no language restriction, and published RCTs that compared efficacy and safety of one kind of disposable device with CC or another device in adult MC were included regardless of whether concealment of allocation or blinding method was carried out or not. The studies including devices that had been confirmed as immature or harmful and thereby not currently used were excluded. Duplicate publications of two or more studies investigating the same sample were excluded.

### Interventions and comparisons

Men were divided into different groups according to the principle of the operation, irrespective of the brand names of devices used. Shang Ring and PrePex were classified as ISD; circular stapler and Unicirc were classified as CDD; and all non-device MCs were classified as CC (e.g., sleeve and dorsal slit). The comparisons were between at least two of ISD, CDD, and CC.

### Outcome measures

The outcomes measured in this review included the following: IB-intraoperative blood loss (ml), OT-operative time (min), PO-mean pain score on the operation day, PP-mean pain score of postoperative days (after 24 h), Com-overall incidence of complication, WAE-incidence of wound adverse event, WB-incidence of wound bleeding, WD-incidence of wound dehiscence, WE-incidence of wound edema, WHT-wound healing time (day), WI-incidence of wound infection, Cost-overall expenditure, SR-satisfaction rate.

### Literature screening and data extraction

Two reviewers (Yu Fan and Dehong Cao) read the titles and abstracts of all potential trials and independently selected eligible RCTs according to the predetermined inclusion and exclusion criteria. If any discrepancy existed, it was resolved by discussion between the two reviewers or by the assistance of a third reviewer (Qiang Wei). Data were extracted and typed in by the first two reviewers through elaborative reading and utilization of a specific formula. The third reviewer (Qiang Wei) checked the information and resolved any differences by discussions with other team members (other authors). If the study provided medians and interquartile ranges instead of means and standard deviations (SDs), we imputed the means and SDs, as described by Hozo *et al.*^30^ In cases of multiple pain scores at different time points, we calculated the mean ± SD pain score to obtain PO and PP. In the presence of missing data, efforts were made to contact the authors for information. Extracted data mainly included general trial information, selected outcomes, and methodological characteristics. The methodological quality of each selected trial was assessed by the same two reviewers according to the Cochrane Collaboration Risk of Bias Tool in Review Manager 5.3. A funnel plot was used to test the possibility of publication bias only when the number of each comparison was no less than ten. Otherwise, the existence of potential publication bias was considered.

### Data processing and statistical analysis

A network plot of devices was drawn in Stata 13.0 to visually display the number of studies involved in each direct comparison. A pairwise meta-analysis was then processed by synthesizing the studies that compared the same interventions with a random-effects model, which incorporated the assumption that different studies assessed different yet related treatment effects^31^. Dichotomous and continuous data were expressed as RR and SMD, respectively, both with 95% confidence intervals (CI).

A network meta-analysis was analyzed in AIDDS v. 1.16.6. Its network meta-analysis models were implemented in the Bayesian framework and estimated by Markov chain Monte Carlo methods. All data processing procedures in AIDDS followed the methods described by Zhao *et al.*^32^ Using the Brooks–Gelman–Rubin diagnostic, we considered that the model had converged when all Markov chains were similar and PSRF was close to 1. Otherwise, additional iterations were applied until the model had converged. In a network meta-analysis, we chose OR and the 95% credibility interval (CrI) to express dichotomous data. This was because logOR was more suitable than logRR as the former had better mathematical properties and often reflected the underlying mechanisms more effectively. Consistency and inconsistency analyses were applied to check whether the trials in the network were consistent. An ICSD greater than RESD indicated an inconsistency problem. Next, we investigated the source for inconsistency from the studies, excluded them, and ran the model until there was no significant inconsistency. A node-splitting model was estimated for each comparison involving both direct and indirect evidence. Whether the evidence was exchangeable was according to the corresponding inconsistency parameter. It should be zero when evidence was completely exchangeable (*p* > 0.05). When there was no relevant inconsistency and evidence was exchangeable, a consistency model was used to draw conclusions on the relative effects (OR, SMD, with CrI) of the included treatments and their rank effects with possibilities. Outcomes were considered to be stable if they were statistically significant in both pairwise and network meta-analysis.

The aforementioned treatment ranks were processed in Stata 13.0 to calculate SUCRA. The larger the SUCRA value, the better recommendation of its best rank, and vice versa. We defined a SUCRA ≥ 80% as a significant recommendation of the best rank and a SUCRA ≤ 20% as a significant consideration of the worst rank.

## Additional Information

**How to cite this article**: Fan, Y. *et al.* The characteristics of circular disposable devices and *in situ* devices for optimizing male circumcision: a network meta-analysis. *Sci. Rep.* **6**, 25514; doi: 10.1038/srep25514 (2016).

## Figures and Tables

**Table 1:** Characters of Included Studies.

Author	Year	Country	Comparison	No. of men	Follow-up(day)	Outcome Measurements
Jin	2015	China	CS vs CC	441/438	90	OT, IB, PO, PP, SR, Cost, WB, WD, WE, WI
Li	2014	China	CS vs CC	129/120	30	OT, IB, PO, PP, PA, WHT, Cost, ARE, WB, WD, WE, WI
Lv	2014	China	CS vs CC vs SR	314/314/314	30	OT, IB, PO, PP, SR, PA, WHT, WB, WD, WE, WI
Wang	2014	China	CS vs CC	60/60	14	OT, IB, SR, WB, WD, WI
Millard	2014	South African	Unicirc vs CC	100/50	28–42	OT, IB, PO, PP, SR, PA, WHT, WB, WD, WI
Kanyago	2013	Uganda	SR vs CC	66/72	21	PO, SR, WHT, ARE, WB, WE, WI
Li	2010	China	SR vs CC	402/322	21	OT, IB, PO, PP, SR, WHT, Cost, WB, WD, WE, WI
Mutabazi	2014	Rwanda	PrePex vs CC	143/73	63	OT, PO, PP, SR, WHT, ARE, WB, WD, WE, WI
Sokal	2014	Kenya, Zambia	SR vs CC	197/201	60	OT, PO, PP, SR, WHT, ARE, WB, WD, WE, WI
Yue	2012	China	SR vs CC	479/354	28	OT, IB, PO, PP, SR, WHT, Cost, ARE, WB, WD, WE, WI

CS: circular stapler, SR: Shang Ring, CDD: circular disposable devices, ISD: *in situ* devices, CC: conventional circumcision, IB: intraoperative blood loss (ml), OT: operational time (min), PO: mean pain score on the operation day, PP: mean pain score of postoperation days, Com: overall incidence of complication, WAE: incidence of wound adverse event, WB: incidence of wound bleeding, WD: incidence of wound dehiscence, WE: incidence of wound edema, WHT: wound healing time (day), WI: incidence of wound infection, Cost: overall expenditure, SR: satisfaction rate.

**Table 2:** Outcomes of Pairwise Meta-analysis

Comparisons	Outcomes	No. of comparisons	RR&SMD	2.5%CI	97.5%CI
**CDD vs CC**	Com	3	0.75	0.21	2.68
	Cost	2	64.1	−2.62	130
	IB*	5	**−3.12**	**−4.32**	**−1.92**
	OT*	5	**−4.33**	**−6.43**	**−2.23**
	PO*	4	**−1.51**	**−2.55**	**−0.46**
	PP*	4	**−1.38**	**−2.28**	**−0.48**
	SR	5	1.12	0.96	1.30
	WAE	5	1.00	0.30	3.39
	WB	5	1.24	0.52	2.97
	WD	5	1.13	0.42	3.03
	WE	3	0.35	0.06	2.02
	WHT*	3	**−0.88**	**−1.18**	**−0.58**
	WI	5	0.67	0.32	1.44
**ISD vs CC**	Com	4	0.87	0.34	2.25
	Cost	2	0.34	−11.5	12.2
	IB*	3	**−3.25**	**−3.65**	**−2.85**
	OT*	5	**−5.72**	**−7.11**	**−4.33**
	PO	6	−1.15	−2.37	0.08
	PP	5	−0.95	−3.09	1.19
	SR*	6	**1.17**	**1.02**	**1.35**
	WAE	6	0.77	0.47	1.26
	WB*	6	**0.16**	**0.03**	**0.76**
	WD	5	1.04	0.66	1.64
	WE*	6	**0.69**	**0.53**	**0.88**
	WHT	6	0.59	−0.11	1.30
	WI	6	0.49	0.12	2.05
**CDD vs ISD**	IB*	1	**0.33**	**0.17**	**0.48**
	OT*	1	**0.48**	**0.32**	**0.63**
	PO*	1	**−2.23**	**−2.43**	**−2.03**
	PP*	1	**−2.39**	**−2.59**	**−2.18**
	SR*	1	**1.57**	**1.39**	**1.78**
	WAE*	1	**0.30**	**0.20**	**0.45**
	WB*	1	**21.0**	**1.24**	**357**
	WD	1	0.63	0.29	1.36
	WE*	1	**0.10**	**0.05**	**0.24**
	WHT*	1	**−0.74**	**−0.90**	**−0.58**
	WI*	1	**0.04**	**0.002**	**0.62**

^*^p < 0.05, RR: relative risk, SMD: standard mean difference, CI: confidence intervals.

**Table 3:** The Outcomes of Network Meta-analysis.

Comparisons	Outcomes	Consistency			Inconsistency		
		OR&SMD	2.5%CrI	97.5%CrI	OR&SMD	2.5%CrI	97.5%CrI
**CDD vs CC**	Com	0.71	0.11	4.46	0.70	0.11	4.49
	Cost*	**245**	**84.1**	**404**	**244**	**76.4**	**407**
	IB*	**−8.24**	**−11.6**	**−4.86**	**−8.40**	**−11.7**	**−4.98**
	OT*	**−17.3**	**−22.6**	**−11.4**	**−17.1**	**−22.9**	**−10.9**
	PO	−1.96	−4.37	0.50	−1.78	−4.25	0.47
	PP	−2.08	−4.74	0.52	−1.80	−4.31	0.65
	SR	2.04	0.21	17.91	1.67	0.22	13.3
	WAE	1.07	0.30	3.99	1.08	0.31	4.15
	WB	2.07	0.18	28.1	2.02	0.19	25.9
	WD	0.97	0.23	3.34	0.98	0.23	3.52
	WE	0.28	0.05	1.59	0.29	0.05	1.68
	WHT	−3.37	−8.16	1.75	−4.04	−8.36	0.80
	WI	0.25	0.02	2.29	0.30	0.03	2.31
**ISD vs CC**	Com	0.84	0.22	3.14	0.84	0.22	3.26
	Cost	8.42	−159	170	9.01	−159	175
	IB*	**−9.26**	**−13.5**	**−4.93**	**−8.50**	**−13.4**	**−3.83**
	OT*	**−18.2**	**−23.7**	**−12.6**	**−18.4**	**−24.5**	**−12.0**
	PO	−1.15	−3.12	0.87	−1.39	−3.35	0.70
	PP	−1.09	−3.74	1.31	−1.64	−3.38	0.78
	SR	5.20	0.72	32.5	**7.85**	**1.02**	**43.6**
	WAE	0.88	0.3	2.73	0.82	0.27	2.57
	WB*	**0.07**	**0**	**0.78**	**0.08**	**0**	**0.89**
	WD	1.14	0.33	3.79	1.07	0.30	4.16
	WE	1.00	0.30	4.75	0.94	0.26	5.39
	WHT	3.26	−0.51	7.05	**4.07**	**0.35**	**7.50**
	WI	0.52	0.07	3.37	0.39	0.06	2.58
**CDD vs ISD**	Com	0.83	0.09	7.71	0.83	0.08	8.05
	Cost*	**235**	**4.91**	**473**	235	−3.19	472
	IB	1.02	−3.99	6.02	2.15	−3.77	8.15
	OT	0.95	−6.23	8.40	0.51	−9.63	11.0
	PO	−0.79	−3.76	2.13	−1.53	−5.08	1.97
	PP	−1.00	−4.43	2.30	−2.55	−6.18	1.61
	SR	0.39	0.03	6.06	1.01	0.04	18.7
	WAE	1.21	0.25	6.08	1.01	0.16	6.64
	WB*	**28.6**	**1.17**	**1320**	**62.8**	**1.44**	**11957**
	WD	0.86	0.15	3.89	0.80	0.12	4.92
	WE	0.27	0.03	1.81	0.24	0.02	2.17
	WHT*	**−6.58**	**−12.6**	**−0.58**	−4.11	−11.1	2.01
	WI	0.47	0.02	7.62	0.24	0.01	5.71

^*^p < 0.05 (in consistency analysis), OR: odds ratio, CrI: credibility interval.

**Table 4:** The Credibility of Network Meta-analysis.

Outcomes	RESD	ICSD	*p*of NS(CDD vs ISD )
**Com**	1.27(0.72,1.67)	0.84(0.04,1.65)	\
**Cost**	73.9(26.5,236)	132(6.15,252)	\
**IB**	3.05(1.66,7.26)	5.13(0.24,13.0)	0.72
**OT**	5.84(3.53,11.8)	8.05(0.31,25.3)	0.97
**IP**	2.19(1.34,3.81)	2.06(0.13,4.16)	0.15
**PP**	2.30(1.34,3.73)	2.50(0.17,3.85)	0.11
**SR**	1.67(0.81,3.60)	2.18(0.07,4.13)	0.14
**WAE**	1.32(0.85,1.68)	0.78(0.03,1.66)	0.22
**WB**	2.27(0.94,3.48)	1.82(0.06,3.46)	**0.03**
**WD**	0.87(0.09,2.20)	0.93(0.04,2.33)	0.80
**WE**	1.29(0.56,2.42)	1.05(0.05,2.45)	0.48
**WHT**	3.47(1.88,6.89)	4.74(0.35,7.92)	0.22
**WI**	1.78(0.87,3.14)	1.75(0.12,3.25)	0.13

RESD: random effects standard deviation, ICSD: inconsistency standard deviation *p* of NS: *p* value of node-splitting models.

**Table 5:** the SUCRA and ranks of treatments.

Methods	Outcomes	SUCRA	Possibilities of ranks		
			Rank1	Rank2	Rank3
**CDD**	Com	61.0	0.51	0.20	0.29
	Cost	**2.0**	0.01	0.02	**0.97**
	IB	65.0	0.30	0.70	0
	OT	69.0	0.38	0.62	0
	PO	**83.5**	**0.72**	0.23	0.05
	PP	**84.0**	**0.73**	0.22	0.04
	SR	48.5	0.21	0.55	0.23
	WAE	43.5	0.31	0.25	0.44
	WB	**14.0**	0.02	0.24	**0.74**
	WD	55.5	0.42	0.27	0.31
	WE	**93.5**	**0.90**	0.07	0.04
	WHT	**95.0**	**0.91**	0.08	0.01
	WI	**81.0**	**0.70**	0.22	0.08
**ISD**	Com	52.0	0.35	0.34	0.31
	Cost	69.5	0.42	0.55	0.02
	IB	**85.0**	**0.70**	0.30	0
	OT	**81.0**	**0.62**	0.38	0
	PO	58.5	0.27	0.63	0.10
	PP	54.0	0.25	0.58	0.17
	SR	**87.0**	**0.77**	0.20	0.03
	WAE	59.0	0.45	0.28	0.27
	WB	**98.0**	**0.97**	0.02	0.01
	WD	40.0	0.26	0.28	0.46
	WE	28.5	0.06	0.45	0.49
	WHT	**3.0**	0.01	0.04	**0.95**
	WI	53.0	0.27	0.52	0.21
**CC**	Com	37.0	0.14	0.46	0.40
	Cost	78.5	0.57	0.43	0
	IB	**0**	0	0	**1.00**
	OT	**0**	0	0	**1.00**
	PO	**8.0**	0.01	0.14	**0.85**
	PP	**11.5**	0.02	0.19	**0.79**
	SR	**14.5**	0.02	0.25	**0.73**
	WAE	47.5	0.24	0.47	0.29
	WB	38.0	0.01	0.74	0.25
	WD	54.5	0.32	0.45	0.23
	WE	28.0	0.04	0.48	0.48
	WHT	52.0	0.08	0.88	0.04
	WI	**16.5**	0.03	0.27	**0.71**

SUCRA: surface under the cumulative ranking curve.
